# Elosulfase alfa enzyme replacement therapy attenuates disease progression in a non-ambulatory Japanese patient with Morquio A syndrome (case report)

**DOI:** 10.1016/j.ymgmr.2017.09.001

**Published:** 2017-09-14

**Authors:** Misako Hiramatsu, Kimitoshi Nakamura

**Affiliations:** a Division of Pediatrics, Nishibeppu National Hospital, Tsurumi 4548, Beppu, Oita 874-0840, Japan; b Department of Pediatrics, Kumamoto University Hospital, 1-1-1 Honjo, Chuo-ku, Kumamoto 860-8556, Japan

**Keywords:** Case report, Elosulfase alfa, Enzyme replacement therapy, Morquio A syndrome, Mucopolysaccharidosis type IV A, Non-ambulatory, ERT, enzyme replacement therapy, GAG, glycosaminoglycan, GALNS, N-acetylgalactosamine-6-sulfatase, KS, keratan sulfate, 3MSCT, 3-minute stair climb test, 6MWT, 6-minute walk test

## Abstract

Enzyme replacement therapy (ERT) with elosulfase alfa is the only approved therapy in Japan for patients with Morquio A syndrome, a lysosomal storage disorder inherited in an autosomal recessive fashion. The experience with ERT in severely affected, non-ambulatory patients has not been reported in previous studies. This case report describes clinical evidence for the 1-year efficacy and safety of ERT with elosulfase alfa in a severely affected, non-ambulatory, 47-year-old patient with Morquio A syndrome who needs intensive respiratory management. ERT with elosulfase alfa was well tolerated in this patient. Because of the possibility of potential hypersensitivity adverse events, special attention is needed when using ERT in patients with respiratory disorders. However, under the appropriate management of specialists, the patient in this case report showed significant respiratory improvement after starting ERT, and abdominal bloating was improved by gas evacuation. In addition, the patient was able to lift up her arms, reach behind her back, and move her legs slightly, and she recovered her grip strength. Her hearing loss improved and she could hear without a hearing aid. This report shows that ERT with elosulfase alfa can be used with appropriate respiratory care in patients with severe respiratory dysfunction.

## 1 Introduction

Morquio A syndrome (mucopolysaccharidosis type IV A; OMIM #253000) is an autosomal recessive lysosomal storage disorder caused by a mutation in the gene for the enzyme N-acetylgalactosamine-6-sulfatase (GALNS, EC 3.1.6.4) located on chromosome 16q24.3. The resulting deficiency in GALNS causes the glycosaminoglycans (GAGs) chondroitin-6-sulfate and keratan sulfate (KS) to accumulate in numerous tissues [1]. Accurate estimates of prevalence and incidence of Morquio A syndrome have not been fully established because of the rarity of the disease and differences in reporting methods. Reported birth prevalence of Morquio A (using recommended diagnostic methods) ranges from 1 per 71,000 in the United Arab Emirates to 1 per 500,000 in Japan [2].

The only approved therapy for patients with Morquio A syndrome is enzyme replacement therapy (ERT) with elosulfase alfa (VIMIZIM^®^, BioMarin Pharmaceutical Inc., San Rafael, USA). In Japan, elosulfase alfa was approved in 2014 based on clinical data from global studies, even though only 6 Japanese patients were included [3]. In most clinical studies used for the new drug application, severely affected patients were excluded. The efficacy evaluation in the clinical studies was mainly assessed by measures of endurance, such as the 6-minute walk test (6MWT) or the 3-minute stair climb test (3MSCT). In these studies, target patients were able to walk at least 200 m [4] or average between 30 m and 325 m in the 6MWT at baseline [5], [6], except for one study in which criteria for enrollment included the inability to walk at least 30 m in the 6MWT at screening [7]. Therefore, ERT experience in severely affected, non-ambulatory patients with Morquio A syndrome is limited.

Here, we present the efficacy and safety of ERT with elosulfase alfa over 1 year in a severely affected, non-ambulatory adult patient with Morquio A syndrome.

## 2 Clinical report

This case report describes a 47-year-old female Japanese patient with Morquio A syndrome. She provided her informed consent for the publication of this case report, including the use of photographs.

The patient was noticed to have delayed growth during infancy. At 5 years of age, she was suspected of Morquio A syndrome based on her short stature, waddling gait, and distinctive skeletal abnormalities. She attended elementary and junior/senior high schools for special needs education. At approximately 20 years of age, she showed scoliosis and hyperlordosis, and worsening valgus deformity of the elbows, ulnar deviation of the wrists, and kyphosis. She used an electric wheelchair for mobility from 23 years of age.

At 34 years of age, she was transferred to Nishibeppu National Hospital with mechanical ventilation after post-influenzal pneumonia led to respiratory failure and impairment of consciousness. At the time of admission, she presented with severe short stature (86.2 cm height), with coarse facial features, hypertrichosis, abdominal bloating, joint contracture, limited finger extension, bone deformity, osteoporosis, slow movement, regression, pain in the limbs and spine, mild cervical myelopathy, cervical instability, hepatic hypertrophy, cardiomegaly, cataract, mild corneal clouding, hearing loss, upper and lower airway narrowing, and respiratory muscle weakness. Her urinary uronic acid concentration upon admission was 6.0 mg/g creatinine (normal range in women aged 20 to 33 years, 3.32 ± 1.26 mg/g creatinine [8]). She was definitively diagnosed with Morquio A syndrome based on her height, the patterns of chondroitin sulfate and KS in electrophoresis [9], and a low galactose 6-sulfatase activity in leukocytes (< 1.0 nmol/mg protein/17 h; N-acetylgalactosamine [GalNAc] 6-sulfatase activity, normal range 187 to 330 nmol/mg protein/17 h [verified by a local laboratory]). She has been confined to bed since admission.

Because of her short stature, short neck, and deformed cervical spine, she was not considered appropriate for weaning off the ventilator after starting spontaneous breathing. She wore a mechanical ventilator after tracheotomy. As a result of cervical myelopathy, she started to show signs of respiratory infection of the bronchus, including progression of bronchial stenosis due to accumulated GAG in the airway and respiratory muscle weakness. She began to develop progressive multiple organ failure due to the systemic lack of galactose 6-sulfatase enzyme activity.

At 35 years of age, the patient experienced hypoglycemia, which was confirmed by blood glucose measurement (22 mg/dL) and treated with glucose-electrolyte (Soldem^®^ 3A) infusions and increased food intake. The cause of hypoglycemia was not determined but was suspected to be related to defective mucopolysaccharide metabolism.

At 40 years of age, stenosis of the bronchial bifurcation, obstruction of peripheral bronchi, wheeze, and airway obstruction were exacerbated due to increased expectoration. She started to use a hearing aid because of worsening hearing loss. Decreased visual acuity and corneal clouding were observed. She was prescribed narcotic analgesics (codeine) for treatment of generalized pain, including back pain, and prednisolone 5 mg/theophylline 160 mg was administered to produce bronchodilation. She began to experience disturbance of consciousness related to hypoglycemia, which occurred in the early morning after overnight fasting and was confirmed by blood glucose monitoring.

At 44 years of age, she received laparoscopic diaphragm plication and gastrostomy at Japanese Red Cross Kumamoto Hospital. After the surgeries, she was transferred to the intensive care unit of Nishibeppu National Hospital for postoperative care. Her atelectasis in the right inferior lobe improved, but pleural effusion and atelectasis remained in the left lung. The patient experienced leakage of enteral nutrition in the gastrostomy site, progressed formation of granulation tissue, and ulceration, and the gastroduodenal tube was replaced. Because of bladder and rectal disturbance, urethral catheterization was carried out. Subsequent observed hypoglycemic episodes were treated by increasing glucose intake (eg, with juice) and prevented by overnight infusions of glucose-electrolyte solution and increased oral glucose intake. Her fasting blood glucose levels were maintained at 60 to 70 mg/dL. The patient was also administered carnitine because of suspected carnitine deficiency.

At 45 years of age, she had significant abdominal bloating due to air swallowing, constipation, and difficulty passing gas. The left diaphragm was weakened, the lung was compressed, and the dyspnea was not improved, despite adjusting the setting of the ventilator. Tube feeding was initiated due to intestinal hypoperistalsis and difficulty with oral feeding. Exacerbated respiratory failure due to bronchitis and signs of heart failure were observed. Her respiratory rate, blood pressure, and pulse were managed by a 24-h monitor.

**Fig. 1:**
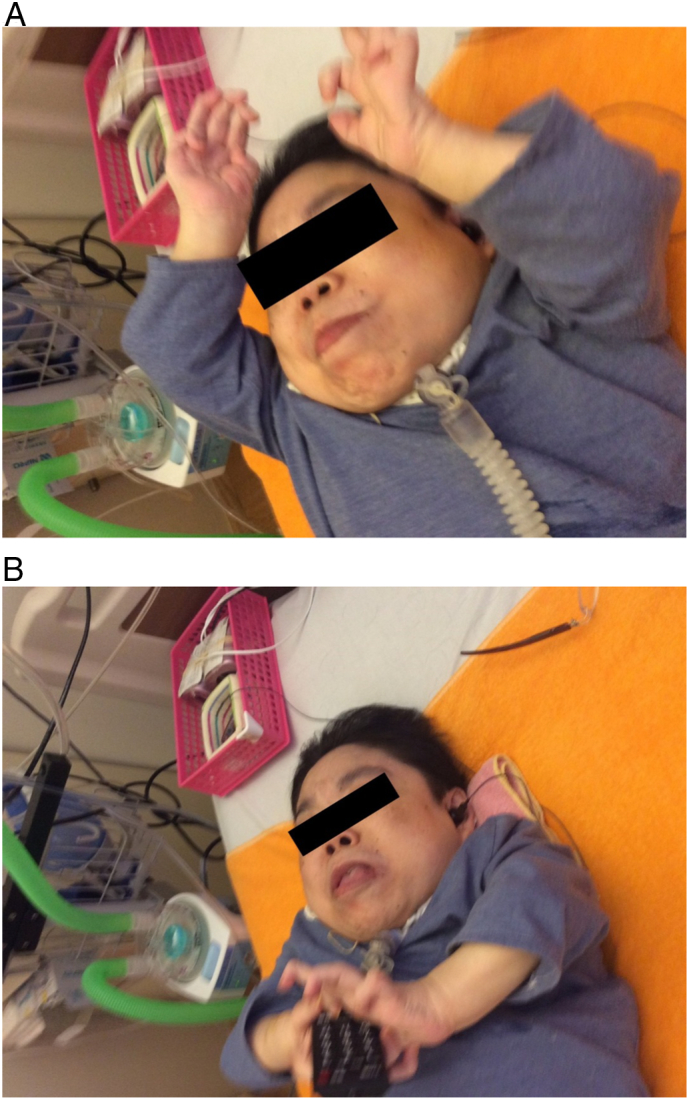
Improvement of hand motion at 1.5 years after starting ERT. A Lift up her arms. B Grip and press a remote control. Abbreviation: ERT, enzyme replacement therapy.

At 46 years of age, she started weekly intravenous infusions of 2.0 mg/kg elosulfase alfa (VIMIZIM^®^, BioMarin Pharmaceutical Inc.). No adverse events related to the ERT were observed after starting ERT. At 6 months after starting ERT, the abdominal bloating was improved in clinical examination and maintained at 1.5 years after starting ERT. Airway resistance in the bronchus, pain of extremities, and corneal clouding were also improved (Table 1). A diminution in the leakage of enteral nutrition in the gastrostomy site was observed, although it was unclear whether this improvement resulted from elosulfase alfa treatment or natural healing. The patient was able to lift up her arms (Fig. 1A at 1.5 years after starting ERT) and reach behind her back, and was able to move her legs slightly. She recovered hand grip strength and was able to grip and press the buttons on a cell phone and remote control (Fig. 1B at 1.5 years after starting ERT). Her hearing improved as she could hear without a hearing aid, and this improvement was confirmed by physicians through conversation with the patient. Hypoglycemia also appeared to improve. These improvements were maintained during ERT, and for 1.5 years after starting ERT, with no safety issues.

**Table 1:** Comparison of clinical signs and symptoms at ERT commencement and thereafter.

Clinical signs and symptoms	At the start of ERT	6 months after starting ERT	14 months after starting ERT
	Aug 2015	Feb 2016	Oct 2016
Respiratory function			
Oxygen flow rate (nasal cannula)	2 L/min	0 L/min (discontinued oxygen inhalation)	0 L/min (discontinued oxygen inhalation)
Proportion of spontaneous respiration	94.71%	98.16%	99.56%
Other important respiratory findings	–	Improved symptom of nocturnal apnea	Improved symptom of nocturnal apnea
Joint pain/fatigue	Severe	No pain/fatigue	No pain/fatigue
Abdominal bloating	Severe	Improved	Improved
Limb motion	Not able to move	Improved, able to move	Improved, able to move
Hearing loss	Exacerbated	Improved, able to hear without hearing aid	Improved, able to hear without hearing aid
Visual loss	Corneal clouding, decreased visual acuity	Improved corneal clouding, recovered visual acuity	Improved corneal clouding, recovered visual acuity

Abbreviation: ERT, enzyme replacement therapy.

## 3 Discussion

This case report provides clinical evidence for the 1-year efficacy and safety of ERT with elosulfase alfa in a severely affected, non-ambulatory adult patient with Morquio A syndrome who needs intensive respiratory management. ERT with elosulfase alfa was tolerated in this severely affected patient and improved her various symptoms, including those related to respiratory function, abdominal bloating, pain in the extremities, corneal clouding, and hearing loss.

In previous clinical studies [4], [5], [6], [7], the efficacy of elosulfase alfa ERT was assessed mainly by measures of endurance, such as the 6MWT, so that most patients needed to have a minimum level of walking ability. As such, non-ambulatory patients with severe symptoms were excluded. However, the observations in this report show that ERT can be safely used in severely affected patients, although appropriate endpoints, not based on walking ability, are needed.

Respiratory impairment is the leading cause of morbidity and mortality in patients with Morquio A syndrome and can be caused by obstructive or restrictive lung disease [7]. Indeed, respiratory failure was the primary cause of death (based on death certificates) in 17 of 27 patients (15 male, 12 female) with Morquio A syndrome between 1975 and 2010 in the United Kingdom [10]. The accumulation of GAG in airway walls, together with thickened secretions and skull, spine, and/or tracheal abnormalities, contributes to narrow and tortuous airways in patients with Morquio A syndrome [1]. Because of the possibility of potential hypersensitivity adverse events, the Japanese package insert for elosulfase alfa warns that more attention is required for use in patients with respiratory disorders [11]. The patient in this case report showed significant respiratory improvement and was able to discontinue supplementary oxygen after starting ERT. In addition, abdominal bloating was improved by gas evacuation, followed by improved lung compression and respiratory function. This report shows that ERT with elosulfase alfa can be used for patients with severe respiratory dysfunction when under appropriate respiratory care.

Hearing loss is one of the most common symptoms found in patients with Morquio A syndrome and may be caused by recurrent respiratory tract infections, otitis media, ossicle deformity, or other inner ear abnormalities [1]. In this report, the observed increase in hearing ability may be explained by an unexpected, direct effect of ERT on hearing and/or an indirect effect of ERT by improved ventilation in the upper airways.

The dose of elosulfase alfa administered to this patient, 2 mg/kg/week, was the approved dose for patients with Morquio A syndrome and was based on the results of clinical study endpoints that evaluated the endurance and safety of ambulatory patients with Morquio A syndrome [5], [12]. Given that elosulfase alfa at 4 mg/kg/week has been shown to have similar safety and tolerability as 2 mg/kg/week, and has also resulted in positive changes in exercise capacity, muscle strength, and pain [4], higher, off-label doses could be considered for severely affected, non-ambulatory patients.

This is the first case report of the ERT elosulfase alfa in a non-ambulatory patient with Morquio A syndrome. There are few reports of ERT experience in Japanese patients, including only 6 Japanese patients prior to the approval of the elosulfase alfa [3]. This report of only one patient limits the ability to apply or interpret these results more broadly. Further, each assessment was performed in a real-world medical setting, resulting in some lack of quantitative data that could otherwise show the extent of improvement. Despite these limitations, this report provides valuable information pertaining to the rare experience of treatment in a severely affected Japanese patient with Morquio A syndrome.

In conclusion, this case report provides clinical evidence for the 1-year efficacy and safety of ERT with elosulfase alfa in a severely affected adult patient with Morquio A syndrome. ERT with elosulfase alfa improved various symptoms, including those related to respiratory function, abdominal bloating, pain in the extremities, corneal clouding, and hearing loss. To manage the diverse symptoms of Morquio A syndrome, it is crucial to administer ERT elosulfase alfa in the setting of multidisciplinary specialist care.

## Funding support

This research did not receive any specific grant from funding agencies in the public, commercial, or not-for-profit sectors.

Medical writing assistance was provided by Hiroko Ebina, BPharm, Ph, MBA and Mark Snape, MB BS, CMPP of ProScribe – Envision Pharma Group, and was funded by BioMarin Pharmaceutical Inc. ProScribe's services complied with international guidelines for Good Publication Practice (GPP3).

## Role of the sponsor

BioMarin Pharmaceutical Inc., manufacturer/licensee of elosulfase alfa, contributed towards the medical writing costs for this report. BioMarin Pharmaceutical Inc. was involved in the decision to submit this case report for publication but did not have a role in the collection or interpretation of data.

## Role of contributors

All authors participated in the interpretation of the patient's course, and in the drafting, critical revision, and approval of the final version of the manuscript. MH, the clinical investigator, was involved in the research design and collected patient data.

## Conflicts of interest

MH and KN have no conflicts of interest to declare.

## References

[1] Hendriksz C.J., Berger K.I., Giugliani R., Harmatz P., Kampmann C., Mackenzie W.G., Raiman J., Villarreal M.S., Savarirayan R. (2015). International guidelines for the management and treatment of Morquio A syndrome. Am. J. Med. Genet. A.

[2] Leadley R.M., Lang S., Misso K., Bekkering T., Ross J., Akiyama T., Fietz M., Giugliani R., Hendriksz C.J., Hock N.L., McGill J., Olaye A., Jain M., Kleijnen J. (2014). A systematic review of the prevalence of Morquio A syndrome: challenges for study reporting in rare diseases. Orphanet J. Rare Dis..

[3] Vimizim (elosulfase alfa) [interview form (in Japanese)]: Tokyo, Japan: BioMarin Pharmaceutical. http://www.info.pmda.go.jp/go/pack/3959417A1027_1_03/, 2015 ((version 2) (accessed 16 Mar 2017)).

[4] Burton B.K., Berger K.I., Lewis G.D., Tarnopolsky M., Treadwell M., Mitchell J.J., Muschol N., Jones S.A., Sutton V.R., Pastores G.M., Lau H., Sparkes R., Genter F., Shaywitz A.J., Harmatz P. (2015). Safety and physiological effects of two different doses of elosulfase alfa in patients with Morquio A syndrome: a randomized, double-blind, pilot study. Am. J. Med. Genet. A.

[5] Hendriksz C.J., Burton B., Fleming T.R., Harmatz P., Hughes D., Jones S.A., Lin S.P., Mengel E., Scarpa M., Valayannopoulos V., Giugliani R., Slasor P., Lounsbury D., Dummer W. (2014). Efficacy and safety of enzyme replacement therapy with BMN 110 (elosulfase alfa) for Morquio A syndrome (mucopolysaccharidosis IVA): a phase 3 randomised placebo-controlled study. J. Inherit. Metab. Dis..

[6] Hendriksz C.J., Parini R., AlSayed M.D., Raiman J., Giugliani R., Solano Villarreal M.L., Mitchell J.J., Burton B.K., Guelbert N., Stewart F., Hughes D.A., Berger K.I., Slasor P., Matousek R., Jurecki E., Shaywitz A.J., Harmatz P.R. (2016). Long-term endurance and safety of elosulfase alfa enzyme replacement therapy in patients with Morquio A syndrome. Mol. Genet. Metab..

[7] Harmatz P.R., Mengel E., Geberhiwot T., Muschol N., Hendriksz C.J., Burton B.K., Jameson E., Berger K.I., Jester A., Treadwell M., Sisic Z., Decker C. (2017). Impact of elosulfase alfa in patients with morquio A syndrome who have limited ambulation: an open-label, phase 2 study. Am. J. Med. Genet. A.

[8] Ida H., Eto Y., Okuyama T., Suzuki Y., Tanaka A., Orii T. (2011). Muko Tatosho UPDATE (in Japanese).

[9] Hopwood J.J., Harrison J.R. (1982). High-resolution electrophoresis of urinary glycosaminoglycans: an improved screening test for the mucopolysaccharidoses. Anal. Biochem..

[10] Lavery C., Hendriksz C. (2015). Mortality in patients with Morquio syndrome A. JIMD Rep..

[11] Vimizim (elosulfase alfa) [package insert (in Japanese)]: Tokyo, Japan: BioMarin Pharmaceutical. http://www.info.pmda.go.jp/downfiles/ph/PDF/641173_3959417A1027_1_03.pdf, 2015 ((version 3) (accessed 16 Mar 2017)).

[12] Hendriksz C., Vellodi A., Jones S., Takkele H., Lee S., Chesler S., Decker C. (2012). Long term outcomes of a phase 1/2, multicenter, open-label, dose-escalation study to evaluate the safety, tolerability, and efficacy of BMN 110 in patients with mucopolysaccharidosis IVA (Morquio A syndrome). Mol. Genet. Metab..

